# Comparative analysis of laparoscopic, retro-muscular, and open mesh repair techniques for ventral and incisional hernias: a comprehensive review and meta-analysis

**DOI:** 10.1007/s13304-024-02049-1

**Published:** 2024-12-20

**Authors:** Amro Elhadidi, Mohamed Shetiwy, Mohammed Al-Katary

**Affiliations:** General Surgery Department, Mansoura Faculty of Medicine, Mansoura University, Mansoura, 35111 Egypt

**Keywords:** Laparoscopic hernia repair, Open repair, Retro-muscular mesh repair, Incisional hernia, Ventral hernia

## Abstract

Ventral hernias are abnormalities in anterior abdominal wall occurring due to an incision or are congenital. This comprehensive review and meta-analysis aim to objectively compare laparoscopic to retro-muscular or any other mesh repair approach to manage ventral incisional hernia. To identify studies that managed ventral incisional hernia using laparoscopic, open, or retro-muscular mesh repair techniques, a comprehensive literature search was performed. Random effects model was used, and data were presented as log odds ratio (logOR) or as Hedge’s *g* with corresponding 95% confidence intervals (CI). Cochran’s *Q* test was implemented to measure heterogeneity among articles, and funnel plots were utilized to examine publication bias visually. Quality of all selected studies was assessed using Critical Appraisal Checklists for Studies developed by the Joanna Briggs Institute. 20 studies (16,247 patients) were included published from 2003 to 2023. Significantly reduced incisional hernias developed in laparoscopic group. The recurrence of hernia lowered with laparoscopic repair vs. open repair. In retro-muscular vs. laparoscopic, recurrence was lower, however, not statistically significant (*p* = 0.97). Open repair type resulted in a longer hospital stay than laparoscopic (*p* = 0.03). In laparoscopic repair, the postoperative complications reduced compared to the open repair (*p* = 0.02). Laparoscopic incisional and ventral hernia repair is a practical and successful alternative to open method. It is associated with shorter hospital stay and lower risk of postoperative complications. In few instances, retro-muscular mesh repair may be opted for.

## Introduction

Ventral hernias are a sort of defect in the anterior abdominal wall. Some other potential causes are congenital (para-umbilical and umbilical) and acquired (incisional) as well. A reported 3–13% of individuals get incisional hernia following abdominal surgery, while postoperative wound infections are observed in up to 20% of patients [1, 2]. Complications include the strangling and ischemia of the hernia contents as well as discomfort, incarceration, and blockage of the intestinal lumen [1, 2].

Ventral/incisional hernias can now be repaired using a variety of approaches. However, there is still debate regarding the best course of action for correcting these hernias, and the recurrence rate is still too high. Despite advancements in healing techniques, there is still a high risk of morbidity and even fatality. Only surgical intervention may be used to make repairs, and two methods are available: open mesh repair and laparoscopic mesh repair [3]. A tension-free repair is possible by inserting a prosthetic material for the reinforcement of the abdominal wall, although such repairs are nevertheless linked to a high recurrence risk of between 2 and 36% [4].

Laparoscopic surgery was developed largely to lessen discomfort through fewer incisions and hasten patient recovery. Numerous randomised controlled trials (RCTs) examined laparoscopic vs. open abdominal procedures, although they mostly assessed short-term results. Earlier systematic reviews concentrated on specific illnesses and surgical intervention types [1, 2, 5]. Due to the infrequent analysis of incisional hernia rates as a result of interest and the short follow-up times, underestimated real rates are expected. Though it is commonly expected that laparoscopic procedures will reduce incisional hernias, there is no quantitative evidence in the surgical literature [5]. The retro-muscular mesh repair is also an excellent and optimal method for treating ventral incisional hernias since it can be used on all incisional hernia sites. The mesh is generally hidden and attached behind the rectus sheath, the risk of complications is low, and the risk of recurrence is also low [2].

The aim of this study is to undertake a meta-analysis for examining the safety and effectiveness of laparoscopic, retro-muscular, and other mesh repairs in ventral hernias. The recurrence rate was the primary outcome, and other considerations were the length of the procedure, the length of hospital stay, and the number of problems during and after surgery.

## Methods

The Preferred Reporting Items for Systematic Review and Meta-Analyses (PRISMA) checklist guidelines were used to conduct the research [6].

### Search strategy

From 2000 to 2023, seven different databases were searched in-depth (Pub Med, Embase, The Cochrane Library, Web of Science, Science Direct, Scopus, and Google Scholar). There were no limitations on the era, the publication language, or the country while doing the literature search. Editorial letters, conference proceedings, and practice recommendations were excluded.

To identify relevant studies, the following key terms were utilized: retro-muscular OR laparoscopic OR “repair” AND “ventral hernia” OR “incisional hernia”. Only research articles were obtained and reviewed. All possible combinations of keywords were utilized.

### Study selection

After removing the duplicates, titles and abstracts were checked for eligibility. We independently evaluated each identified abstract’s full-text article.

### Criteria for considering studies

The inclusion criteria comprised published studies (randomized trials and large prospective and retrospective cohorts) reporting retro-muscular versus laparoscopic mesh repair of ventral hernia. The exclusion criteria include (1) studies without results; (2) studies not related and not providing sufficient data (3) studies reporting other types of hernias (4) case reports, guidelines, commentaries, editorials, letters to the editor, book chapters, reviews and meta-analysis (5) studies that utilize other minimally invasive hernia repair techniques (6) studies in languages other than English.

Additionally, the references of earlier systematic reviews and meta-analyses were checked for pertinent studies.

### Data extraction and outcomes of interest

Data were obtained from certain investigations by the two independent reviewers. Discussion was used to settle any disputes. For data extraction, a typical Excel spreadsheet was used. Table 1 lists the general information and study characteristics that were considered. For each included study, the year of publication, authors, country, patient characteristics (age, defect size, surgical technique), study design, and effectiveness outcomes (hernia recurrence, operative time, postoperative complications, and length of hospital) were collected.

**Table 1:** Baseline characteristics of studies included in the meta-analysis

Author	Year	Country	Study design	Sample size	Mesh type	No. of patients	Mean Age	Defect area (cms)	Operation time (min)	Postoperative complications	Hospital stay (days)	Recurrence	Risk of bias assessment
Ahonen-Siirtola M [8]	2015	Finland	Retrospective comparative study	891	Open	291	61	41	121	68	6	8	Moderate
					Laparoscopic	527	59	31	105	97	4	22	
Asencio F [9]	2021	Spain	Follow up study of an RCT	85	Open	39	60	10.2	NA	0	NA	9	Moderate
					Laparoscopic	46	58	9.51		3		9	
Bellido Luque J [10]	2021	Spain	Case–control study	79	Endoscopic retro-muscular	40	60.1	62.9	106.8	6	1.3	0	Moderate
					Laparoscopic	39	54.9	57.3	61.4	19	1.8	1	
Bui NH [11]	2022	Denmark	Retrospective cohort study	72	Extraperitoneal retro-muscular	29	57	9.1	103.4	6	0	5	Low
					Laparoscopic intraperitoneal	43	57	11.8	82.4	7	1	4	
Christoffersen MW [12]	2023	Denmark	Retrospective cohort study	59	Retro rectus	27	57	9	117.2	1	0	NA	Low
					Laparoscopic intraperitoneal	32	54.5	6.2	84.4	6	1		
Earle D [13]	2006	USA	Prospective cohort study	426	Open	158	53	NA	89	1	2	27	Moderate
					Laparoscopic	268	51		149	3	1	9	
Ecker BL [14]	2016	USA	Retrospective cohort study	13,567	Open	9228	58.8	NA	NA	175	3	1245	Moderate
					Laparoscopic	4339	59.1			39	2	386	
Eker HH [15]	2013	Netherland	RCT	194	Open	107	56.7	25	76	26	3	14	Low
					Laparoscopic	99	59.5	25	100	35	3	17	
Goh SSN [16]	2023	Singapore	Retrospective cohort study	174	Open	86	62.5	61.6	116	13	NA	9	Moderate
					Laparoscopic	88	61.5	60	139	1		10	
Itani KM [17]	2010	USA	RCT	146	Open	73	59.6	45.9	127	35	3.9	6	Low
					Laparoscopic	73	61.2	45.7	155	23	4	9	
LeBlanc KA [18]	2021	USA	Prospective cohort study	371	Robotic-Assisted	159	59.7	49.8	126.2	16	1.4	0	Low
					Open	130	59.1	57.5	107.1	7	2	1	
					Laparoscopic	82	55.6	56.2	57.2	7	1.4	0	
McGreevy JM [19]	2003	USA	Prospective cohort study	136	Open	71	55.8	NA	102	15	1.5	NA	Moderate
					Laparoscopic	65	53.8		132	5	1.1		
Qadri SJ [20]	2010	India	Prospective cohort study	80	Open	40	35.5	55.2	90.3	10	4.33	1	Moderate
					Laparoscopic	40	33.6	62.2	75.1	2	1.53	1	
Raakow J [21]	2018	Germany	Prospective cohort study	28	Open	20	58.8	11.3	168.1	4	11.5	0	Moderate
					Laparoscopic	8	60.1	9.8	96.1	2	7	3	
Rogmark P [22]	2013	Sweden	RCT	133	Open	69	58	25	110	29	2	0	Low
					Laparoscopic	64	58	36	100	21	2	0	
Warren JA [23]	2016	USA	Prospective cohort study	156	Robotic-Assisted	53	52.9	82.5	245.6	5	1	2	Moderate
					Laparoscopic	103	60.2	88	121.5	7	2	2	
Tsuruta A [24]	2014	Japan	Retrospective cohort study	45	Open	21	67.4	8.9	152.7	6	13.4	1	Low
					Laparoscopic	24	69.8	8.7	143.1	3	6.8	0	
Yang S [25]	2022	China	Retrospective cohort study	155	Open	82	63.2	NA	76.7	14	13	7	Moderate
					Laparoscopic	73	60.8		63.6	44	6.9	15	
Zolin SJ [26]	2019	USA	Retrospective cohort study	186	Open-retro	105	57	6	NA	16	3	1	Moderate
					Laparoscopic	81	55	5		16	1	2	
Demetrashvili Z [27]	2017	Georgia	RCT	155	Retro-muscular	77	59.6	100.4	155.1	17	5.2	2	Low
					Onlay	78	61.2	92.7	124.5	39	5.5	4	

### Study quality assessment

Using the Critical Appraisal Checklists for Studies [7] from the Joanna Briggs Institute (JBI), each selected study’s quality was assessed according to the study design. For studies with a “yes” score of 49% or below, the risk of bias in the study was deemed to be high. The risk of bias was considered moderate for studies with a score of 50–69%, and studies with a 70% score or more had a low risk. All the studies included were evaluated for the risk of bias and then classified accordingly, i.e., studies with low risk, high risk of bias, and studies with some concerns. Discussion and consensus were used to resolve disagreements between the two independent reviewers.

### Statistical analysis

STATA version 17 was used to carry out this meta-analysis. Continuous data were expressed using means, medians, and relevant standard deviations or ranges. Additionally, for descriptive purposes, categorical variables were shown as percentages and integers. There was a pooled meta-analysis. To evaluate the heterogeneity between studies, Cochran’s *Q* test was implemented based on the approach described by DerSimonian and Laird. High heterogeneity was defined as an *I*^2^ value of more than 50%, moderate heterogeneity between 25 and 50%, and low heterogeneity as less than 25%. All the variables were analyzed using a random effect model. Using funnel plots, publication bias was visually investigated. Statistics were considered substantial if the *p* value was 0.05 or below.

## Results

### Identification and description of studies

**Fig. 1:**
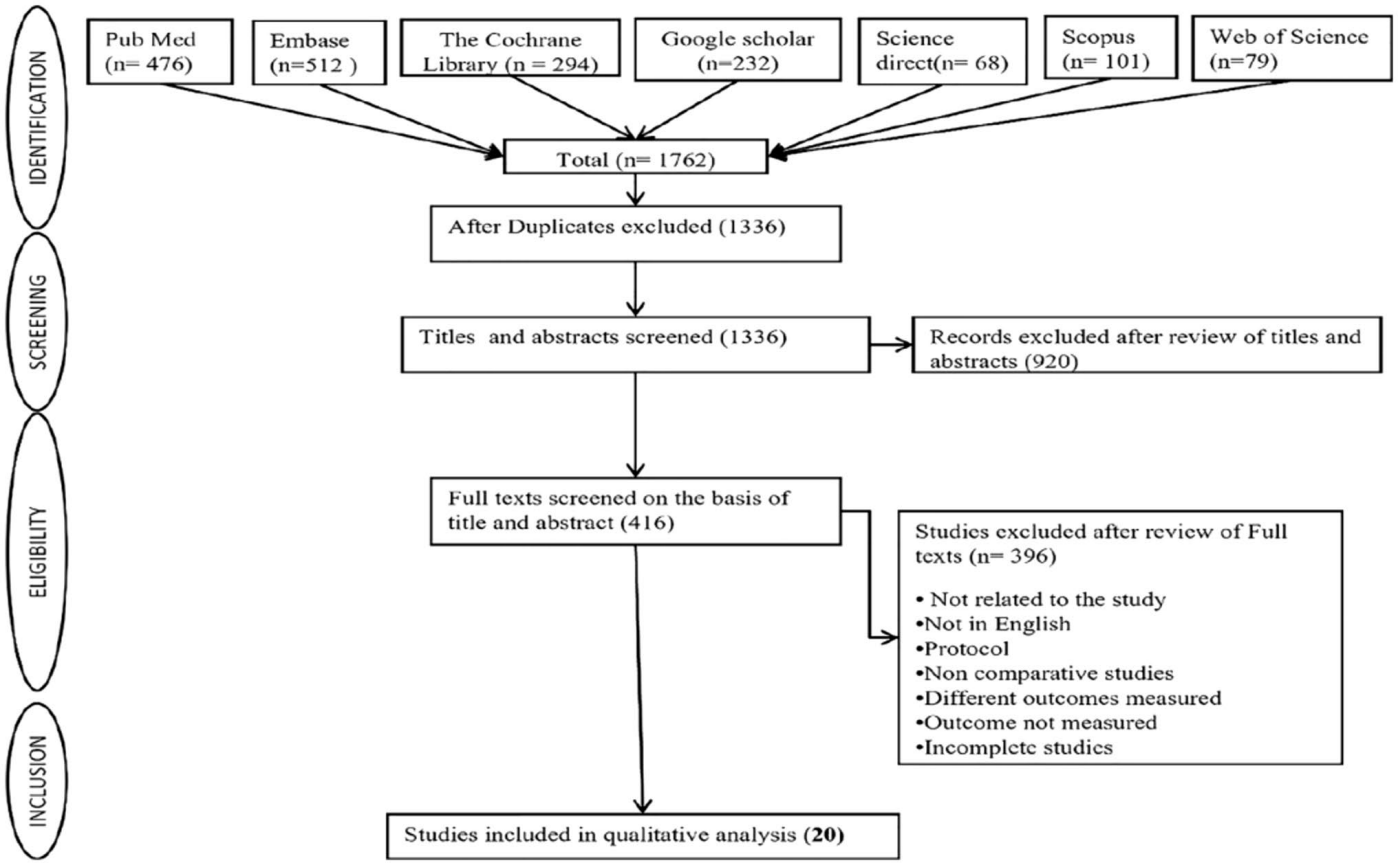
Flowchart showing the process of selecting or rejecting articles to include in the study

1762 citations were identified in total, of which 426 duplicate studies were eliminated. These included 476 from PubMed, 512 from Embase, 294 from The Cochrane Library, 232 from Google Scholar, 101 from Scopus, 68 from Science Direct, and 79 from Web of Science. 920 studies were excluded after evaluating the titles and abstracts of 1336 articles. The remaining 416 articles met the requirements for the full-text review. After applying exclusion criteria, 396 complete texts were eliminated, leaving 20 articles to go through the final qualitative analysis. How the study selection procedure was carried out is given in the flow diagram (Fig. 1). The 20 publications included 5 RCTs, 1 case control study, 6 prospective cohort studies, and 8 retrospective cohort studies.

### Characteristics of the included studies

An overview of the major clinical and demographic characteristics of each included research is given in Table 1 [8–27]. 16,247 patients took part in the research overall. The years of publication varied from 2003 to 2023, and the sample size was between 28 and 13,567. Out of the 20 published studies, 7 were conducted in the United States, 2 in Denmark, 2 in Spain, and 1 each in Germany, Sweden, Georgia, Japan, Netherlands, Singapore, Finland, India, and China.

### Clinical outcomes

#### Primary outcome: rate of recurrence

**Fig. 2:**
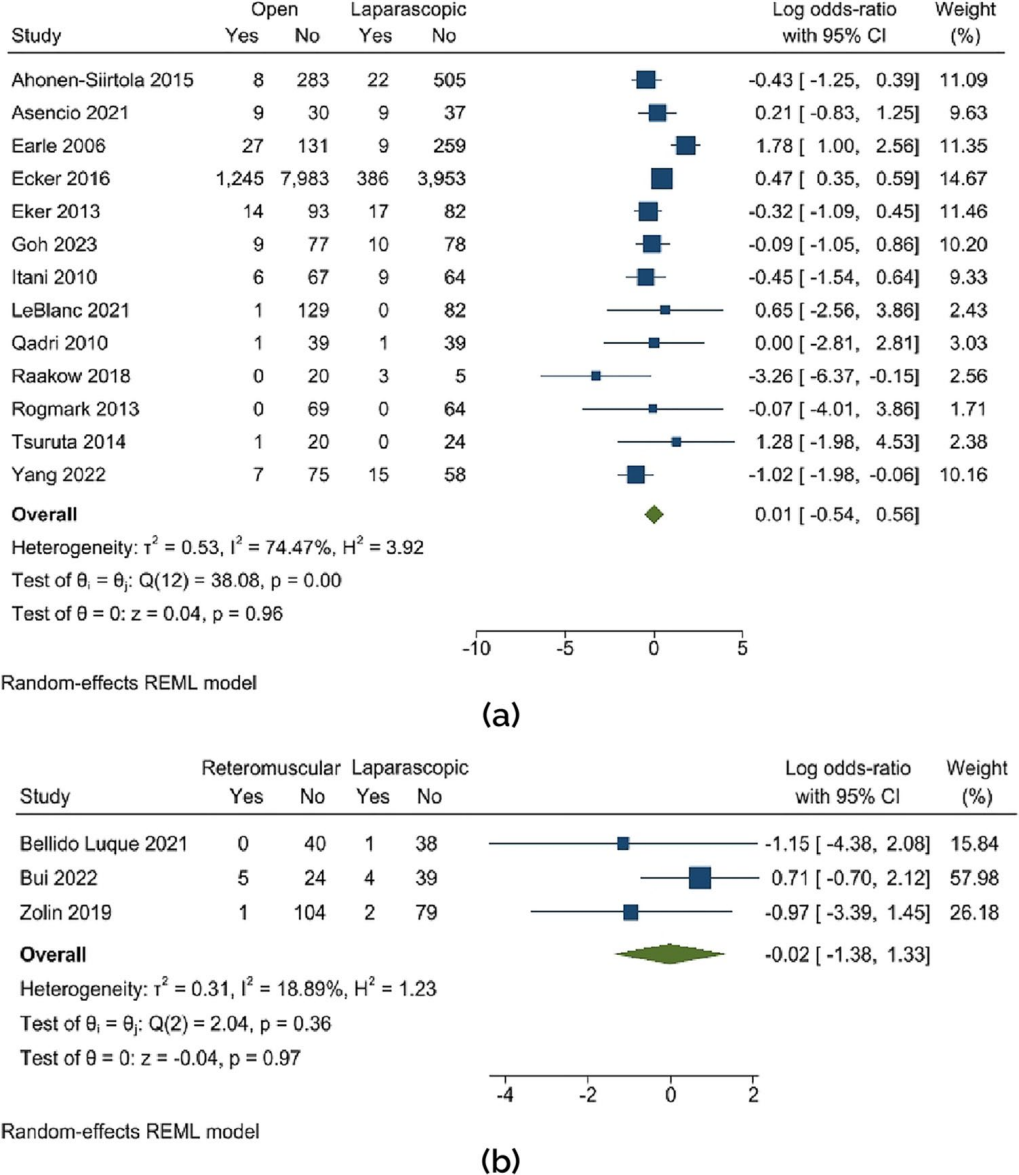
Recurrence **a** open vs. laparoscopic, **b** retro-muscular vs. laparoscopic mesh type

A meta-analysis assessed the prevalence of ventral hernias using data from 20 studies involving 16,247 subjects. There is a pattern that suggests there was a decreased overall rate of hernia recurrence after laparoscopic treatment compared to open repair (OR 0.01; 95% CI − 0.54 to 0.56; *p* = 0.96), and the substantial heterogeneity still remained (*Q* 38.08; *p* < 0.000). In the case of retro-muscular versus laparoscopic, the recurrence was lower in the retro-muscular mesh type, although not statistically significant (OR − 0.02; 95% CI − 1.38 to 1.33; *p* = 0.97). Refer to Fig. 2.

#### Operative time

**Fig. 3:**
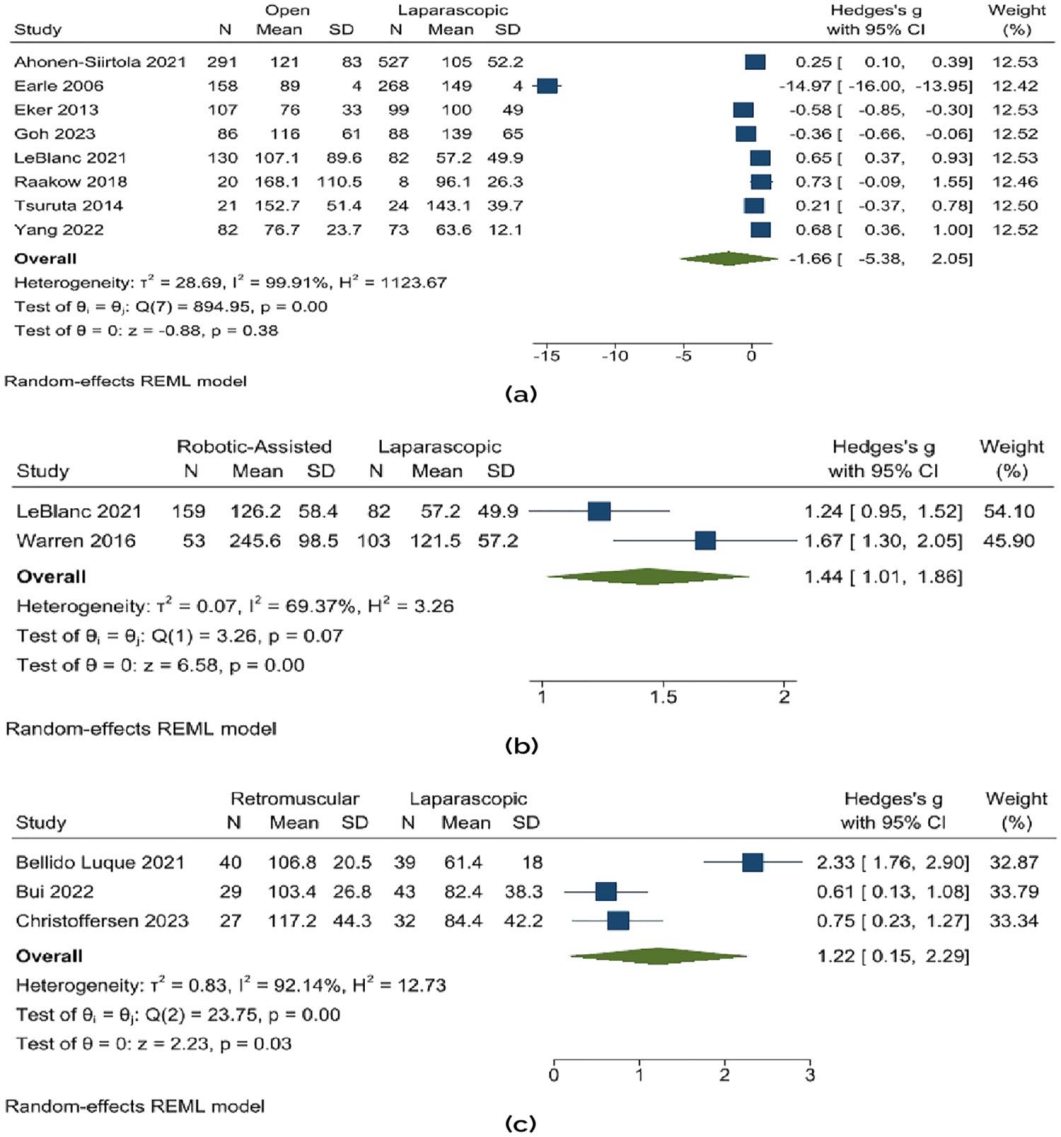
Duration of operation (minutes) **a** open vs. laparoscopic, **b** robotic-assisted vs. laparoscopic, **c** retro-muscular vs. laparoscopic

The fixed effects model was inadequate since there was high heterogeneity among the studies. Figure 3 illustrates how open mesh repair takes more time compared to laparoscopic repair. This difference was statistically insignificant (random effects model: Hedge’s *g* − 1.66; 95% CI − 5.38 to 2.05; *p* = 0.38). However, the operative time difference between a robotic-assisted and a laparoscopic repair was significant (Hedge’s *g* 1.44; 95% CI 1.01–1.86; *p* = 0.000). The difference in the operative time between a retro-muscular repair and a laparoscopic repair (Hedge’s *g* 1.22; 95% CI 0.15–2.29; *p* = 0.03) was also substantial. Figure 3 shows this.

#### Defect area

**Fig. 4:**
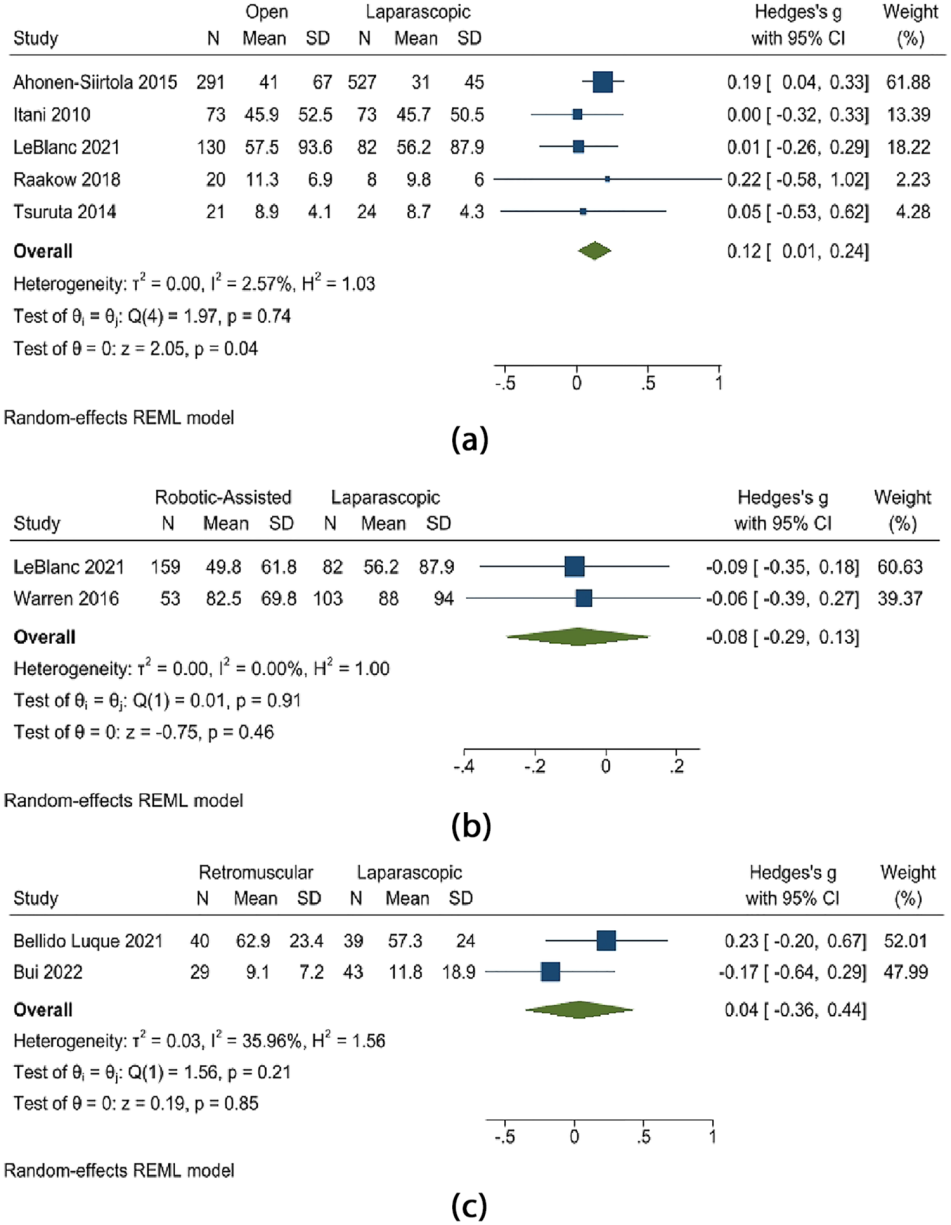
Defect area (cm^2^) **a** open vs. laparoscopic, **b** robotic-assisted vs. laparoscopic, **c** retro-muscular vs. laparoscopic

The defect area (cms) was larger in open mesh repair than in laparoscopic repair (random effects model: Hedge’s *g* 0.12; 95% CI 0.01–0.24; *p* = 0.04). There was, however, no substantial difference in the defect areas when laparoscopic repair was compared to robotic-assisted repair and retro-muscular repair. This is shown in Fig. 4.

#### Postoperative complications

**Fig. 5:**
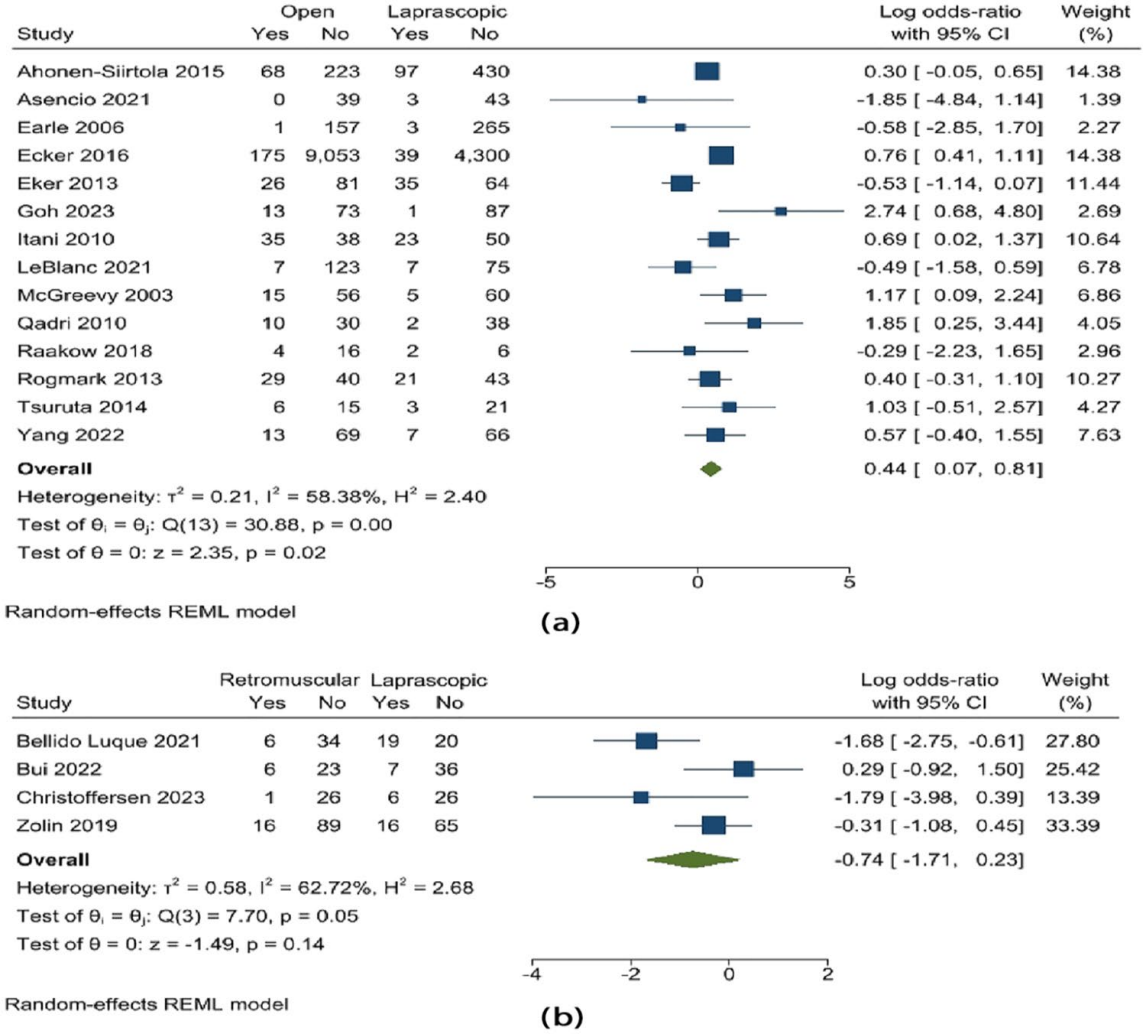
Postoperative complications **a** open vs. laparoscopic, **b** retro-muscular vs. laparoscopic

According to a study of the composite variable of postoperative complications that took into account the various outcomes (random effects model: OR 0.44; 95% CI 0.07–0.81; *p* = 0.02), a reduced incidence of postoperative problems were observed in the laparoscopic repair of a hernia. However, the postoperative complications in retro-muscular mesh repair were lower than in laparoscopic repair, although insignificant. Figure 5 shows this.

#### Length of hospital stay

**Fig. 6:**
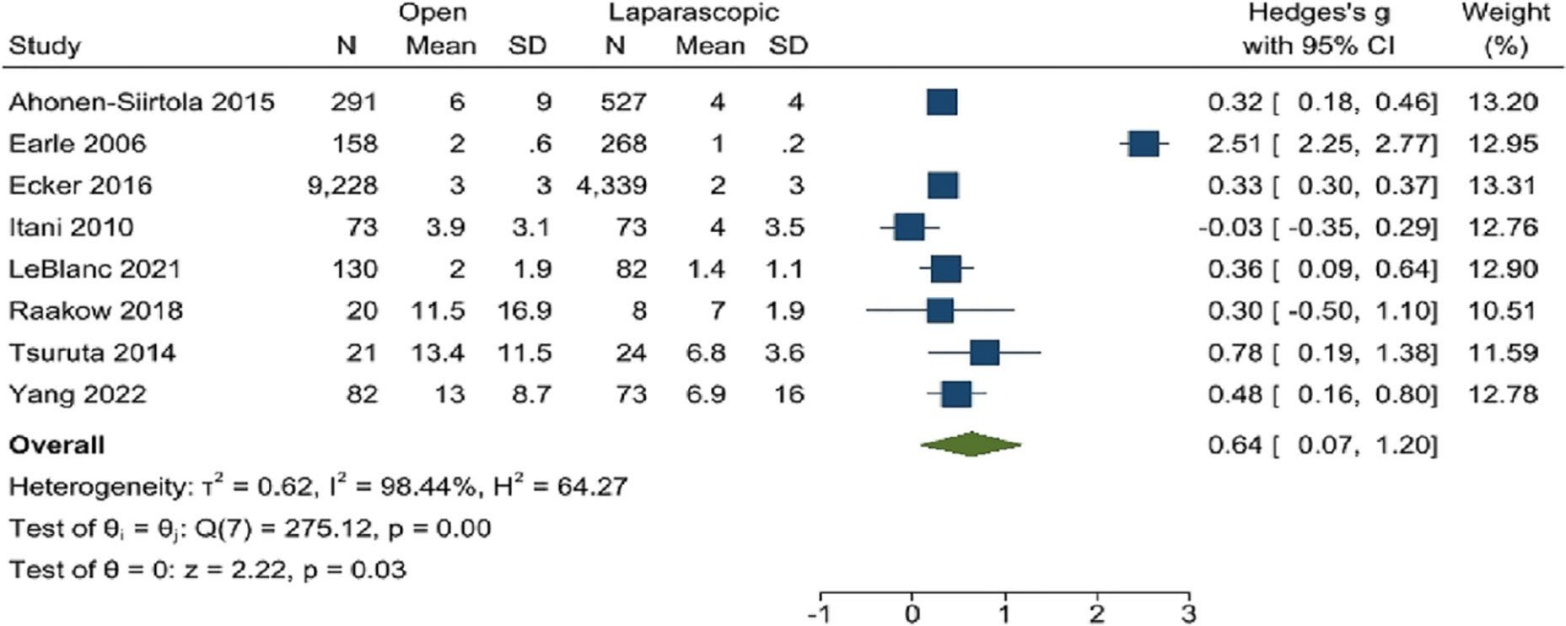
Length of hospital stay (days)

The length of hospital stay in the included studies was compared after laparoscopic versus open mesh repair. Open mesh repair type showed a considerably longer hospital stay than laparoscopic (random effects model: Hedge’s *g* 0.64; 95% CI 0.07–1.20; *p* = 0.03, *I*^2^ = 98%). The result analysis showed that laparoscopic repair was linked to a considerably shorter hospital stay. Figure 6 shows this.

### Heterogeneity

The most common methods for detecting heterogeneity in meta-analysis include *I*^2^ Index and *Q* test. An *I*^2^ score of 0% indicates no between-study variability is present in the analysis and that all variances are the product of sampling error. On the other hand, the closer an *I*^2^ index gets to 100%, the more the observed variance may be attributed to between-study variability rather than just sampling error. Most of the outcomes in the studies included showed significant heterogeneity in this meta-analysis.

### Study quality assessment and publication bias

Each included study’s quality was evaluated independently by two reviewers. A low-to-moderate risk of bias existed in most included studies in this study. The majority of the funnel plots show asymmetry, implying that publication bias exists for most results. To sensitively identify publication bias, however, there were insufficient studies for all these characteristics.

## Discussion

This is the first comprehensive evaluation and meta-analysis that, to our knowledge, compares LAP with other surgical approaches. 20 studies comparing the laparoscopic method to the open, retro-muscular, or robotic-assisted approaches involved 16,247 patients in total.

Ventral hernias are described as the protrusion of a part of a tissue or an organ via a weakness in the abdominal wall. Following abdominal wall surgery, this hernia incidence might reach 13%. An incisional hernia following a procedure on the abdominal wall is considered morbidity. These hernias are more likely to occur when certain risk factors are present, including wound infection, obesity, male sex, abdominal distension, occasionally inadequate surgical closure, and underlying disease processes. Nearly 350,000 ventral hernia repairs (VHR) are thought to be done yearly in the USA alone, at a cost of over $3 billion [28]. Previous studies have shown that VHR may contribute to total cost losses for healthcare systems [28].

In an effort to improve the value of care, surgeons have looked for ways to decrease the risk of hernias in patients undergoing initial abdominal operations as well as to lower intraoperative costs, improve patient outcomes, and shorten the length of stay (LOS) for patients undergoing VHR [26]. Incisional hernia incidence after midline laparotomy varied from 11 to 20% following a mean follow-up of between 12 and 20 months. After incisional hernia repair, incisional hernias have a significant recurrence risk. After open mesh surgery, the recurrence rate of hernia varied from 6.8 to 32.1%, while following laparoscopic repair, it ranged from 0 to 12.3%. Due to the high likelihood of recurrence following incisional hernia repair, both anatomical and prosthetic repairs have been tried. With a high recurrence frequency of roughly 30–50% following anatomical repair and 1.5–10% following prosthetic mesh repairs, the outcomes have been dismal. With the notion of tension-free mending, prostheses have transformed hernia surgery. Even though many different surgical techniques have been used to treat incisional hernias, prosthetic mesh implantation is still the most effective treatment option. Both the preperitoneal plane formed between the rectus muscle and posterior rectus sheath (sublay mesh repair) and the subcutaneous abdominal wall tissues and the anterior rectus sheath (onlay mesh repair) are suitable locations for the prosthetic mesh. The latter method has a number of benefits, one of which is that it rests relatively deeply in the preperitoneal plane and prevents infection from spreading from subcutaneous tissues down to the mesh [5].

Using a prosthetic mesh in a tension-free repair is the gold standard for treating ventral/incisional hernias. The open repair of a hernia may now be performed with this technique, or as an alternative, a laparoscopic approach can be used [29]. In comparing mesh-based repair to suture-based repair, an analysis of several incisional hernia repair approaches along multiple outcome characteristics suggests that mesh-based repair gives a superior choice. The main issue is the cost involved and the increased danger of infection brought on by the implantation of a foreign body. The considerable dissection and tissue manipulation involved in hernia surgery have been widely blamed for postoperative problems, including seroma development, hematoma, cellulitis, and wound infection [1, 28]. Mesh repair has a higher intraoperative blood loss rate and longer operating duration. These two elements have reportedly been linked to a rise in wound infection [1]. A large overlap of around 5 cm over the hernia defect in all directions with the mesh positioned in the preperitoneal, retro-muscular region was first used in the late 1980s. When this technique was improved, the recurrence rates dropped to as low as 3.5%, and it was deemed the standard of treatment for ventral hernias [1, 28].

The basic tenets of the open retro-muscular (preperitoneal) repair that Stoppa and Rives describe, which call for positioning the mesh in this plane, offer several benefits [25]. Furthermore, since the mesh is retro-muscular in a deeper plane, any infection in the subcutaneous plane has no effect on it because of how highly vascular this plane is. The preperitoneal method enables a uniform distribution of pressures throughout the mesh's surface area. This explains the robustness of the repair and the related reduction in recurrence. The posterior rectus sheath is attached to by the prosthesis, rendering it inextensible and preventing additional herniation, dislodging, or rupture due to intra-abdominal pressure. Instead, the force that produced the hernia holds the prosthesis in place [25, 28].

The laparoscopic intraperitoneal onlay mesh (IPOM) technique quickly gained popularity after the first report of laparoscopic ventral hernia repair published by LeBlanc et al. in 1993 since it resulted in a quicker recovery and less severe wound problems [18]. They provided evidence of the benefits of laparoscopic hernia repair over open surgery, with better outcomes and reduced complication rates. With the full loss of abdominal muscular structure, the only condition currently regarded as unfavourable for a laparoscopic technique is a major tissue defect. However, despite the fact that the hernia repair procedure has evolved in the last 20 years with regard to general methodology, many specialists still find the results to be subpar. Recurrence rates for incisional hernias treated with a primary suturing range from 12 to 54%, whereas they might reach 36% with mesh repairs. Furthermore, introducing foreign bodies like the Prolene mesh can have major negative effects like discomfort, fistula, infection, bowel adhesions, and bowel damage. The production aspects of the most recent mesh product types have been given greater thought in order to minimise the issues outlined above. Since that time, laparoscopic repair has been frequently used like a reliable alternative for open hernia surgery [2, 25].

The minimally invasive laparoscopic method involves a few stab-like incisions to allow the usage of laparoscopic equipment. Instead of fixing the fascial defect, the procedure involves covering it with mesh, either with or without shrinking the hernia sac. For a safe procedure with fewer risks, such as seroma, infection, haemorrhage, and intestinal damage, a thorough dissection is essential. According to some publications, laparoscopic incisional hernia repair yields improved results compared to open surgery since the recurrence rate is much lower (4.3%), and there are fewer wound complications [3]. A large overlap of around 5 cm over the hernia defect in all directions with the mesh positioned in the preperitoneal, retro-muscular region was first used in the late 1980s. It was deemed the standard of therapy for ventral hernias as the refining of the procedure reduced recurrence rates to as low as 3.5% [28].

Laparoscopic incisional hernia repair has purportedly demonstrated improved results with respect to postoperative complications and hernia recurrence. Wound complication rates can be as low as 38%, and recurrence rates can be as low as 43% during a 25-month follow-up period, according to a meta-analysis of several non-randomized comparative studies and case series. The laparoscopic approach provides minimally invasive access to the abdominal cavity. It allows for the placement of a prosthetic deep inside the abdominal tissue. A laparoscope is utilized to view the abdominal cavity and assist in the placement of the prosthesis after making several tiny incisions in the abdominal wall far from the hernia. Often, the hernia sac is left alone, and sutures, tacks, or a combination of the two are used to anchor the mesh in place. With this approach, the surgical insult is reduced. Additionally, it makes the defect more visible, including any secondary hernia flaws that are smaller and might not be seen clinically. As a result, the prosthesis may be placed more precisely with consistent fascial overlap. The limited amount of dissection needed might also lessen the chance of problems, including infection, gut wall damage, bleeding, and seroma development [28]. There were fewer wound infections with laparoscopy, according to a recent meta-analysis of ten published randomised, controlled studies comparing the open to laparoscopic technique [30]. Even without taking into account patient advantages like early hospital release and return to work, cost–benefit analyses have shown laparoscopic incisional hernia surgery to be equally expensive as open incisional hernia repair [3].

Surgery is required to treat an incisional hernia in 80% of patients. More than 20% of open incisional hernia repairs result in morbidity, including mesh infection and recurrence. Surgery is required to treat an incisional hernia in 80% of patients. More than 20% of open incisional hernia repairs result in morbidity, including mesh infection and recurrence. In general, laparoscopic surgery is risk-free and linked to fewer infections and a shorter hospital stay. Due to the incisional hernia's good exposure in obese individuals, it is quite practical. The surgical treatment, however, might be challenging, and it can take more time. It has not yet been determined which course of action is ideal for treating incisional hernias. A more well-defined treatment plan may have advantages, including a shorter hospital stay, lower costs, and fewer postoperative problems [31]. According to two recent meta-analyses, laparoscopic repair is as successful as open surgery and may even be superior in some cases [1, 28]. Less postoperative problems were seen, and the overall hospital stay was shorter.

According to a recent meta-analysis, incisional hernias following midline abdominal incisions are anticipated to occur 13% of the time at 2 years following the first operation, and approximately 80% of patients would ultimately need another procedure. Since its original description in 1993, laparoscopic incisional ventral hernia repair (LVHR) has been proven to be both secure and effective. Additionally, several surgical teams have begun inserting intraperitoneal mesh through trocar ports to address abdominal wall defects. Recent systematic reviews support the LVRH approach’s effectiveness and safety, but they also highlight the dearth of research that looks at the quality of life over the long term [8, 28].

Laparoscopic and open repairs for hernias did not vary in hernia recurrence, according to a meta-analysis. In six of the eight included trials, though the hospital stay length was shorter after laparoscopic surgery, the difference in operation time remained ambiguous. Fewer wound infections were linked to laparoscopic hernia repair [28]. Incisional hernias were substantially less common in the laparoscopic group, according to another meta-analysis. Additionally, the laparoscopic group’s wound infections were much lower than those in the open group, and both groups’ total postoperative morbidity was comparable. Compared to laparoscopy, open abdominal surgery resulted in a noticeably longer hospital stay [5]. According to Zhang et al. [2], investigations revealed that the laparoscopic group had a shorter hospital stay duration than the open group, and wound infection occurrences were considerably lower in the laparoscopic group compared to the open group (laparoscopic group 2.8%, open group 16.2%). Al Chalabi et al. compared laparoscopic versus open abdominal incisional hernia repair for effectiveness and safety [3]. They found that the recurrence rate was comparable, the length of hospital stay was not statistically different, wound infection was higher in the open repair group, and laparoscopic surgery took more time. Sains and colleagues examined data from 351 patients who were participants in five studies and discovered that the laparoscopic procedure resulted in a 12 min longer operating time that was and a 3–3 days shorter hospital stay; no difference was there in the wound infection rates or hernia recurrence rates [32]. One-fourth of mesh repairs and nearly half of all initial repairs fail after three years, according to a randomised, multicentre research. Additionally, using prosthetic materials increases the chance of serious wound complications, which in and of itself is a risk factor for hernia recurrence and additional morbidity [4].

The included studies included a substantial amount of heterogeneity, which was one of the study’s limitations. Due to the fact that none of the included studies were double-blinded, research bias is the first potential factor contributing to heterogeneity. Confounding factors, such as various hernia locations and sizes, patients with various surgical risks, and various meshes, are the second potential reason for heterogeneity. The fact that there was a sizable difference in operating methods between the trials is a second restriction.

While our systematic review compares laparoscopic IPOM procedures to open retro-muscular approaches, we recognize that recent advances in hernia surgery, including as laparo-endoscopic and robotic-assisted techniques, have moved the field's emphasis. However, most of the papers that satisfied our inclusion criteria focused on standard laparoscopic IPOM and open retro-muscular methods. This limitation reflects the condition of the literature at the time we conducted our review.

We understand that comparing IPOM to retro-muscular techniques may no longer adequately convey the intricacies of modern hernia repair strategies. As such, the findings given here should be taken into consideration the existing evidence. Future research is needed to make a more thorough comparison of emerging procedures, such as laparo-endoscopic and robotic retro-muscular repairs, with open repairs. This will be critical for guiding future clinical practice and improving surgical techniques.

## Conclusions

This meta-analysis led us to the conclusion that laparoscopic repair of ventral hernias is a feasible, safe, and efficient alternative to open repair methods. It is linked to a reduction in perioperative problems, shorter hospital stays, and maybe quicker procedures. As a result of its universal applicability to all incisional hernia sites, the mesh’s anchoring behind the rectus sheath, low complication rate, and low recurrence rate, we also noted that a retro-muscular mesh repair is a good approach for treating ventral incisional hernias. Because of the methodological and clinical heterogeneity observed in this research, high-quality studies with larger patient groups are required to support the conclusions of this thorough analysis.

## Data Availability

Not applicable.
